# Fear of COVID-19, Mental Health and Resilient Coping in Young Adult Male Inmates: A Portuguese Cross-Sectional Study

**DOI:** 10.3390/ijerph20085510

**Published:** 2023-04-14

**Authors:** Rute Mendes, Wanessa Cristina Baccon, Carlos Laranjeira

**Affiliations:** 1 Estabelecimento Prisional de Leiria, Avenida da Comunidade Europeia, No 1-Apartado 460, 2410-755 Leiria, Portugal; 2 School of Health Sciences of Polytechnic, University of Leiria, Campus 2, Morro do Lena, Alto do Vieiro, Apartado 4137, 2411-901 Leiria, Portugal; 3 Postgraduate Nursing Program, State University of Maringá, Av. Colombo, 5790-Zona 7, Maringá 87020-900, PR, Brazil; 4 Centre for Innovative Care and Health Technology (ciTechCare), Rua de Santo André—66–68, Campus 5, Polytechnic University of Leiria, 2410-541 Leiria, Portugal; 5 Comprehensive Health Research Centre (CHRC), University of Évora, 7000-801 Évora, Portugal

**Keywords:** mental health, pandemic, inmates, fear or COVID-19, coping, Portugal

## Abstract

Inmates are highly exposed to mental and physical disorders. Therefore, periodic screening of their mental health and other health risks is required. This study aims to investigate the perceived fear of COVID-19 and the psychological impact of the pandemic in a sample of young adult male inmates. An institutional-based quantitative cross-sectional study design was performed. Data collection took place from July to September 2022 at a juvenile prison in the central region of Portugal. Data were collected using questionnaires on demographic and health characteristics; fear of COVID-19; depression, anxiety and stress levels; and resilient coping. The sample included 60 male inmates imprisoned for over 2 years. Stress was the most common symptom among inmates (75%), followed by anxiety (38.3%) and depression (36.7%). The mean score on the Fear of COVID-19 Scale was 17.38 ± 4.80, indicating relatively low fear levels. Low resilient scores were found in 38 participants (63.3%). Participants reported moderately high ranges of 3.62 ± 0.87 regarding mental health perception, 3.73 ± 0.95 for physical health perception, and 3.27 ± 0.82 for global health concerning the previous month. The Pearson correlation matrix indicated significant and moderate to strong correlations between fear of COVID-19 and mental health-related variables (*p* < 0.001). The predicting factors of fear of COVID-19 were identified by deploying a multiple linear regression model. We found four predictors: age, perception of mental health, and overall levels of anxiety and stress (R^2^ = 0.497). Fear of a given scenario or factor may shift with time. Therefore, long-term research is needed to evaluate whether fear of COVID-19 is adaptive or long-reactive in victims. Our study can assist policymakers, mental health and public health experts, and others in recognizing and managing pandemic-related fears and mental health symptoms.

## 1. Introduction

COVID-19 is an infectious disease caused by SARS-CoV-2 [1] that represents a health emergency for prisons and custodial settings worldwide. Presently, the world prison population exceeds 10 million, in most cases with poor hygiene and unhealthy conditions [2,3]. The risk of infection among jailed persons is 5.5 times greater than in the general public [4]. Several variables contribute to this increased risk, including the higher chronic disease burden of the inmate population [5] and the architecture of prison institutions, which hinders common preventative tactics, such as social isolation from one another and from prison staff [6].

Prisons are an essential component of international public health preparedness and response to COVID-19, as they can act as reservoirs leading to the reactivation of the virus in the general population [7]. The prison context is often identified as an overcrowded, hostile environment with high aggression and instability and very specific organizational dynamics. The response capacity of prisons was tested by the COVID-19 pandemic. Preventive measures, such as environmental restrictions and the absence of significant social contact, negatively affected psychological well-being and maintenance of hope during imprisonment and increased the likelihood of anxiety, psychological distress, and other negative emotional responses after release [8,9]. 

The World Health Organization (WHO) released recommendations on infection prevention and control in prisons [10], emphasizing the need for social distancing, personal protective equipment, and inmate mental health care [3], and also the use of throat swabs and serological tests to detect COVID-19 infection among inmates and prison workers (police officers, physicians, nurses, administrators, and so on). Finally, immunization may be an effective method to minimize the spread of COVID-19 infection in the prison population [3].

In addition to concerns about the disease itself and public health, several authors have alerted to the effects on mental health during the COVID-19 pandemic [11]. Fear elevates stress and anxiety levels in healthy people and exacerbates these symptoms in those with psychiatric conditions [12]. Fear is described as an unpleasant emotional state caused by the perception of potentially dangerous stimuli [13], such as those brought about by a pandemic [14]. Psychological well-being is affected by the fear of infection by a possibly lethal, fast-spreading virus, the origins, nature, and course of which are unknown [15,16]. Furthermore, increased constraints within jails may exacerbate threats to inmates’ human rights [17]. 

Recent mental health research discovered a higher prevalence of psychopathology in prison/correctional settings, such as anxiety, depression, paranoia, psychosis, suicidal behavior [18], and substance use [19,20]. Considering the pooled prevalence of mental illness in prisons, treatment barriers appear to increase the poor response to this problem. Indeed, past research has shown that incarceration creates specific mental health care challenges [21]. Often, these constraints begin with the arrival of prisoners, who are often reluctant to seek aid on their own. Screening at reception usually emphasizes physical health, while mental health screening is frequently limited. Furthermore, screening is frequently performed by workers who have no prior training in mental health issues [21], contributing to the inefficiency of such evaluations.

In “normal conditions,” the prison population presents high levels of mental and emotional vulnerability, a situation that has worsened due to the COVID-19 pandemic [21]. As a result of their uncertain and high-risk circumstances, inmates suffer increased degrees of anxiety, fear, and stress [16]. However, few national studies have portrayed their mental health status in the context of the COVID-19 pandemic, thus justifying the present study.

### Research Problem

In 2020, there were 11,424 prisoners in Portugal, which means a prison rate of 111 prisoners per 100,000 inhabitants [22]. The incarcerated population is “dominated by men, people from disadvantaged socioeconomic groups, with lower cultural and educational levels, and often from ethnic, religious, or political minorities” [23] (p. 4). In Portugal, the introduction of special procedures for prison release, as well as the temporary suspension of activities and prison visits, helped avoid and mitigate the transmission of the virus, with no documented COVID-19-related deaths. Nonetheless, the confinement measures had a negative influence on the mental health of individuals in jail and their families, with the greatest impact on incarcerated juveniles [24].

According to the transactional model of stress and coping proposed by Lazarus and Folkman, people constantly assess stimuli in their surroundings and cope with stress [25] using different strategies to manage emotions or address the cause of the problem [26]. Fears of infection, along with limits on family visits and worry about delays in an already overburdened court system, have heightened mental health risks [27] related to increased rates of anxiety, stress, and depression [16,21]. Moreover, the perception of poor social support may lead to the adoption of maladaptive coping strategies [28].

During times of uncertainty and stress, resilient coping provides a counterweight to personal and social events that promote anxiety. Evidence suggests a relationship between resilience and task-based stress coping [29]. Positive emotional expression is one way resilient people adapt mentally to difficult situations. Ensuring a connection with family and friends through letter writing and video conversations has been shown to be a highly effective means to increase the quality of support. Furthermore, in-cell activities—such as physical activity, mindfulness exercises, puzzle games, drawing, videos, and playing cards—provide creative alternatives to boredom and lack of social interactions and help boost optimal mental health and well-being [16]. Sorge et al. [30] also highlight hope and gratitude as positive emotional support from prison workers. 

There is a paucity of understanding about the impacts on the mental health of inmates during the COVID-19 pandemic. More research is needed to identify risk and protective variables and quantify their prevalence. This might potentially guide policies and intervention initiatives to improve mental health care services in prison settings. This study aims to investigate the perceived fear of COVID-19 and the psychological impact of the pandemic on a sample of young adult male inmates. 

Specifically, we aimed: (1) to characterize the sociodemographic and health variables of a sample of inmates; (2) to determine their mental health status, perceived fear of COVID-19, and resilient coping; (3) to examine the relations between fear of COVID-19, mental health symptoms, and resilient coping; and (4) to identify predictive factors associated with fear of COVID-19. 

## 2. Materials and Methods

### 2.1. Study Design

An institution-based quantitative cross-sectional study design was carried out and reported in compliance with STROBE (Strengthening the Reporting of Observational Studies in Epidemiology) checklist [31].

### 2.2. Setting and Sample 

Data for this study were collected in a juvenile prison (a penitentiary institution intended for the confinement of people in preventive detention but also convicted persons) located in the central region of Portugal. This is the only juvenile prison in Portugal with inmates aged between 16 and 25 who are deprived of their liberty while serving their sentence or pre-trial measure. The causes of detention include violence, crimes against property, and crimes related to the consumption of narcotics. As a prison for young people, inmates are encouraged to join occupational activities that help them pass the time in seclusion, but that, above all, will be crucial for a good reintegration into the community after their release [32]. 

Participants were selected using a convenience sampling technique. Potentially eligible individuals were included in the study if they: (a) were adults (minimum of 18 years of age); (b) could speak and write in Portuguese; (c) were sentenced and in prison before the pandemic COVID-19 began; and (c) presented the adequate intellectual and cognitive ability to understand the nature of the research. Participants who did not meet the inclusion criteria were excluded. 

### 2.3. Data Collection

Self-reported data were obtained in person between July and September 2022. The questionnaires were adjusted to fit the participants based on the findings of a pretest and opinions from experienced psychologists and police officers from the penitentiary. One trained mental health nurse who worked in the detention center conducted the distribution and collection of questionnaires. However, during data collection, the nurse wore regular clothes (not a uniform), signaling that data gathering was not part of and would not affect their prison trajectory. Completion of the survey lasted about 10 min. Out of 150 potential subjects, 60 inmates were accepted to participate and enrolled (response rate of 40%). Completed questionnaires were promptly collected and verified by the principal researcher to detect overlooked items and prevent missing data.

### 2.4. Instruments

Data were collected through a paper survey which includes four parts:

(1) Personal information covered sociodemographic variables (age, education, marital status, nationality, length of imprisonment, and employment status before arrest); health information (history of chronic illness [Yes/No/Unsure] and the presence or history of SARS-CoV-2 infection [Yes/No]); and perception of global, physical, and mental health through a scale from 1 (bad) to 5 (excellent), with reference to the previous month.

(2) Depression Anxiety and Stress Scale (DASS-21 [33], Portuguese validation of Apóstolo et al. [34]) is a set of three sub-scales (i.e., depression, anxiety, and stress) designed to measure negative emotional symptoms. Each sub-scale consists of 7 items rated on a 4-point Likert scale from 0 (“did not apply to me at all”) to 3 (“applied to me very much”) [33]. Before interpreting the results, the summed scores in each sub-scale were multiplied by 2. The total scores were interpreted as follows: “DASS-Depression: normal (0–9), mild (10–12), moderate (13–20), severe (21–27), and extremely severe (28–42) depression. DASS-Anxiety: normal (0–6), mild (7–9), moderate (10–14), severe (15–19), and extremely severe anxiety. (20–42). DASS-Stress: normal (0–10), mild (11–18), moderate (19–26), severe (27–34) and extremely severe stress (35–42)” [35] (pp. 3–4). In this study, Cronbach’s alpha values were 0.811, 0.765, and 0.840 for stress, anxiety, and depression sub-scales, respectively.

(3) Fear of COVID-19 Scale (FCV-19S [14], Portuguese validation by Soares et al. [36]) assesses the fear triggered by the new coronavirus. It is a unidimensional self-response scale composed of seven items that assess participants’ level of agreement using a 5-point Likert scale (“1—Strongly Disagree and 5—Strongly Agree”) [14]. There are no reverse-scored items. The global score ranges between 7 to 35, with a higher score indicating a greater fear severity [14]. Cronbach’s alpha for the FCV-19S in this sample was 0.839.

(4) Brief Resilient Coping Scale (BRCS [37], Portuguese validation of Pais Ribeiro and Morais [38]) is a unidimensional self-report scale comprising four items that aim to understand the ability to deal with stressful situations in an adaptive way. Responses are given on an ordinal scale, in Likert format, with five positions (5-Almost always, 4-Very often, 3-Often, 2-Occasionally, 1-Almost never). The global score varies between 4 and 20. According to the authors of the original scale [37], subjects with a score lower than 13 are considered to have low resilience, and those with scores greater than 17 have strong resilience. Cronbach’s alpha value for this scale was 0.785, which denoted an acceptable internal consistency [39].

### 2.5. Ethical Considerations

The Local Ethical Review Board (CE/IPLEIRIA/30/2022) approved the study protocol. Participation was anonymous and voluntary. After being fully informed about the nature of the study, all participants provided written informed consent. Respondents were expressly informed that they might withdraw from the research at any moment and stop answering any questions that made them uncomfortable. Throughout the data-collecting procedure, all information received from participants was anonymized and kept anonymous. There were no monetary rewards for completing the survey.

### 2.6. Data Analysis

All data analyses were carried out using SPSS 28.0 software (SPSS Inc., Chicago, IL, USA). Descriptive statistics (e.g., frequencies, percentages, range, means ± SD [standard deviations]) were used to describe the sample characteristics. The Shapiro–Wilk test was used to assess data normality. Pearson correlation (r) was used to evaluate the statistical association between fear of COVID-19, depression, anxiety, stress, and resilient coping. Subsequently, a multivariable logistic regression model was used to investigate the factors – such as sociodemographic factors, health information, health-related perceptions, resilient coping, and mental health issues (independent variables)—that might predict fear related to COVID-19 (dependent variable). Sociodemographic variables were introduced first, then variables measuring health information and health-related perceptions, and finally, psychological and mental health variables were introduced. All models were adjusted for sociodemographic variables (age, education, professional situation, and nationality). We used Variance Inflation Factors (VIFs) to analyze the multicollinearity of covariates. The multiple logistic regression included only factors with VIFs less than 2.0. The level of significance was set at *p*-value < 0.05.

## 3. Results

### 3.1. Sociodemographic Characteristics and Health Information of Participants 

The details regarding the personal characteristics of the participants appear in Table 1. The average age was 21.9 years (ranging between 18 and 25). Among the 60 inmates, 66.7% were of Portuguese nationality, 48.3% had completed the 3rd cycle, 38.3% were students before imprisonment (38.3%), and all surveyed individuals had been imprisoned for more than 2 years. A high percentage indicated they had no medical comorbidities (86.7%). Around 43% referred either currently had or had a history of SARS-CoV-2 infection. 

### 3.2. Mental Health Status, Fear, Coping, and Health-Related Perceptions among Inmates

Stress was the most common symptom among inmates (75%), followed by anxiety (38.3%) and depression (36.7%) (Table 2). Low resilient scores were found in 38 participants (63.3%). Participants reported moderately high ranges of 3.62 ± 0.87 regarding mental health perception, 3.73 ± 0.95 for physical health perception, and 3.27 ± 0.82 for global health concerning the previous month. Most considered their global health as very good (42.4%), their physical health as very good (33.9%), and their mental health as good (47.5%). The mean score of the inmates on the Fear of COVID-19 Scale was 17.38 ± 4.80, indicating relatively low fear levels. 

**Figure 1:**
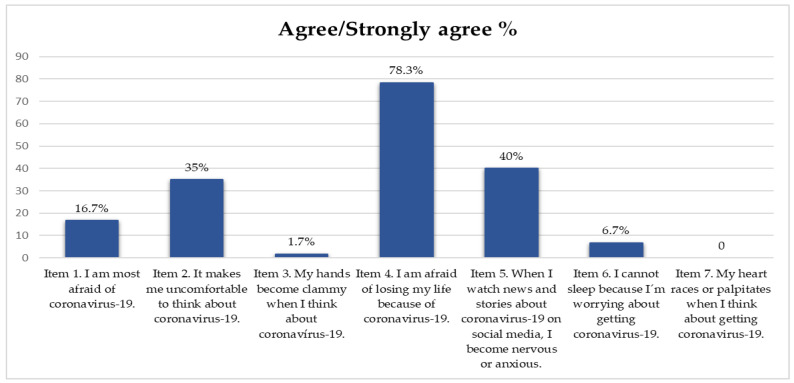
Inmates’ agreement on the seven items of FCV-19S.

Participants’ agreement with the seven FCV-19S items is shown in Figure 1: 16.7%, 35%, 1.7%, 78.3%, 40%, 6.7%, and 0% of inmates agreed with being scared, uneasy, clammy, fearful of losing life, anxious or nervous, unable to sleep, and feeling palpitations due to COVID-19, respectively.

### 3.3. Correlation Analysis between Study Variables 

Pearson correlation analyses were used to test bivariate associations between study variables (Table 3). Examination of the correlation matrix indicated significant and moderate to strong positive correlations between fear of COVID-19 and every mental health variable, but none of these variables indicated significant multicollinearity (*r* > 0.90) [40]. Fear of COVID-19 was not significantly correlated with resilient coping.

### 3.4. Predictive Factors of Fear of COVID-19

Table 4 shows the predictors of fear of COVID-19 based on hierarchical multiple regression models adjusted by selected control variables. Sociodemographic variables entered the first model, wherein age accounted for 7% of the variance in fear of COVID-19. In the second model, the perception of mental health showed statistical significance in predicting fear of COVID-19 for an additional 20.2% of the variance. The final model included mental health status and resilient coping, controlling for the other variables. This increased the variance explained by 22.4%. The results revealed that fear of COVID-19 was positively related to age, anxiety, and stress but negatively associated with the perception of mental health. The final model was able to explain 49.7% of the total variance in fear of COVID-19, with mental health-related predictors explaining most of the variance.

## 4. Discussion

To the best of our knowledge, our study is the first survey of the mental health of inmates in Portugal during the COVID-19 pandemic. According to our findings, 75%, 38.3%, and 36.7% of individuals, respectively, exhibited mild to extremely severe stress, anxiety, and depression symptoms. Recently published studies found that being incarcerated is consistently related to an increased risk of poor mental health [41,42]. Furthermore, we found higher levels of depression and anxiety than those indicated by research on depression worldwide – where depression was 33.7%, and anxiety was 31.9% [43]—and lower than among Iranian and Ethiopian prisoners, where depression varied between 44% and 66.4% and anxiety between 56.3% and 66.9% [44,45]. Our results fall within the spectrum of a recent review where the burden of depression among prisoners ranged between 35.3 and 38.0% [46]. Similarly to Birkie et al. [45], variation in our findings might be due to the global burden of COVID-19 during our study, the sampling method, the response rate, the instruments used, and the setting under study. 

Our data stress that inmates experienced both high mental health challenges and low rates of resilient coping (63.3%). Other studies reveal that “individuals with lower psychological resilience levels were easy to suffer from negative emotions such as anxiety and depression” [47] (p. 2). Given these findings, it is critical to continue studying this prisoner population to understand better the protective factors of resilience (e.g., self-acceptance, family functioning, and social support) that may mitigate the consequences of the COVID-19 pandemic. Recent evidence has also “shown that resilience increases well-being, eliminates the symptoms of anxiety and depression, and increases self-esteem, gratitude, optimism, and mental well-being” [29] (p. 3).

Reasons for stress and anxiety include uncertainty, fear of infection, psychological distress, and anguish, all elements exacerbated during the pandemic [48,49]. The pandemic also promoted suffering in detainees, including substance misuse, risk of self-harm, and suicidal ideation [50]. Other factors were also identified, such as health anxiety, i.e., “when observed bodily sensations (connected to infectious disease or not) are perceived as an illness” [49] (p. 106). If a person constantly feels anxious or experiences stress for a long time, their body no longer receives signals to return to normal functioning. This can weaken a person’s immune system and make them more vulnerable to viral infections and common diseases. Moreover, “the juvenile justice population has profound mental health morbidity, often related to previous trauma, that may become exacerbated by fear, social distancing, and disruptions in care, housing, schooling, and routine because of COVID-19” [51] (p. 2). 

Overall, participants perceived their global, physical, and mental health as good or very good. This result is relevant “as greater self-perceived health has been associated with a greater experience of depression, anxiety, and psychological distress in various populations” [52] (p. 6). This means that worse self-perceived health may be associated with poor mental health. Indeed, this strengthens the need for more research on protective factors to enhance the likelihood of positive outcomes.

In our study, inmates experienced varying levels of fear across the seven items of the FCV-19 scale. While research on inmates’ fear of COVID-19 is still limited, our findings are consistent with previous studies that indicate fear, psychological impacts, and uncertainty owing to COVID-19 [16,18]. The intensified fear of COVID-19 among detainees indicates that adequate attention to their psychological needs is required during unprecedented periods such as the ongoing pandemic. Therefore, one of the priorities when managing the pandemic should be addressing the psychosocial and mental health issues of inmates. In such an unstable situation, social relationships are critical for the physical and emotional well-being of inmates.

This study identified that inmates with the worst mental health indicators (stress, anxiety, and depressive symptoms) were more fearful than those with better status. Fear of COVID-19 is expected to cripple people as they become increasingly anxious about its harmful impact, causing psychological suffering [53]. Previous research has revealed that fear of the unknown is a basic emotion and a core component in the maintenance of anxiety [54]. COVID-19-related concerns encapsulate not only fear of the unknown but also increasing existential threat and anxiety associated with unexpected and unmanageable circumstances [55]. Fear is also associated with sadness and anxiety [14]. Satici et al. [56] confirmed that the unreadiness to handle ambiguity and uncertainty is connected to pandemic-related fears through perseverative cognition (rumination) and that the dominant negative mood harmed well-being.

We also identified variables that are statistically significant in predicting fear of COVID-19. The multivariate analysis indicated that fear of COVID-19 seems to be related to age, perception of mental health, stress, and anxiety. Fear of COVID-19 increased with age and depression and anxiety symptoms but decreased with a higher perception of mental health. Interestingly, higher awareness of mental health predicted lower levels of fear, which might lead to consistently practicing preventive behaviors [57]. Many studies have also found that age has an impact on COVID-19 fear levels [58,59]. The link between affective symptoms and fear of COVID-19 might be explained by catastrophizing, a negative thought pattern of anticipating the worst possible outcome in a particular scenario. This cognitive distortion is connected with anxiety and depression. During the COVID-19 crisis, there was a gloomy and chaotic view of the world, accompanied by unpleasant feelings and emotions involving significant levels of fear and loneliness. Our findings suggest that negative patterns of thinking warp reality and may lead to intensified fear of COVID-19 and emotional dysregulation, such as depressive and anxiety symptoms [60,61].

The current research has potential implications for policy, research, and practice. First, promoting mental health programs and services must be a government priority as an integral element of the COVID-19 response [62]. These initiatives must be multifaceted, integrating efforts to safeguard and enhance mental health with activities to manage other mental illnesses [62]. Second, when inmates enter the penal system and serve their sentences, they should be screened for mental health disorders on a regular basis [21]. Third, this study may help develop psychotherapeutic programs to evaluate how the incarcerated might best cope with stress and manage emotions [41]. In terms of therapeutic approaches, cognitive-behavioral techniques (e.g., cognitive restructuring) may be beneficial by lowering negative thoughts, worry, and anxiety, which in turn may lead to excessive fears. Inmates can become more confident and literate in mental health by combining psychoeducation and psychosocial support oriented toward fear and stress [53]. More research is urgently needed, not just to acquire a comprehensive knowledge of the mental health effects in detention centers but also to design and run complex interventions to improve inmate health. Given the likelihood of another pandemic, person-centered planning and competent mental health services are necessary to foster meaningful connections that will enhance well-being and reduce fear [63]. Long-term planning and crisis response during pandemics require further research and resources to lessen the stress and psychological burden on inmates. Finally, the COVID-19 pandemic deepened “pre-pandemic pains of imprisonment, increasing the ‘tightness,’ ‘depth’ and ‘weight’ of participant’s time in custody” [64] (p. 218). There is widespread agreement that jails and penitentiaries are often ill-equipped to meet the needs of inmates with mental health concerns [65,66]. Offering tele-mental health services might be an excellent strategy to meet the requirements of inmates while also boosting rapport building based on mutual respect and trust [65].

### Study Strengths and Limitations

Various limitations to this study should be mentioned. First, the cross-sectional design lacks stable and causal evidence for discovered associations. With longitudinal designs, the factors investigated can be better understood. Second, this study relied heavily on self-reported questionnaires to assess mental symptoms and made no clinical diagnoses. Third, the sample was not drawn at random and was modest in size. Fourth, this study only included inmates who were in prison before the beginning of the pandemic, thus excluding individuals that confronted the pandemic before their arrest and incarceration and probably presented different psychological and mental states. Finally, because males in prison are heterogeneous (in terms of age, demographic factors, criminal characteristics, and similar characteristics), it is challenging to extrapolate current outcomes to other populations of jailed men. Furthermore, data were collected over the 3rd trimester of 2022, when fear and worry about COVID-19 were less pervasive in social media and the public consciousness than during the onset of the pandemic and, therefore, less likely to trigger mental health problems. Finally, fear of a certain event or element might shift over time [67]. Long-term research is needed to discover whether COVID-19 fear is adaptive or long-reactive in victims.

Despite the above limitations, the current study has some strengths, namely (a) the use of widely used and standard self-reported measures, (b) the effort to study mental health problems in prison settings, and (c) giving helpful information to assess and support the most vulnerable young inmates [30]. Moreover, favorably or neutrally phrased questions elicit genuine replies from research subjects. These measures may have avoided the effects of social desirability in this study. Finally, the study emphasizes the role of resilient coping as a positive variable that may help to adopt positive coping styles and promote mental health. Although the causality/relationship between resilient coping and other mental health variables was not confirmed, it is imperative to collect prospective data and consider protective factors during public health planning in prisons.

## 5. Conclusions

Our study reveals a high prevalence of stress among inmates, as well as moderate levels of anxiety and depression. Likewise, some predictive variables significantly related to fear of COVID-19 were identified, namely: age, perception of mental health, and overall levels of anxiety and stress. Early detection and treatment of disruptive psychopathological symptoms among prison inmates must become a common practice. Different stakeholders (e.g., prison staff, healthcare professionals, and public health officials) should address the risk factors in order to support and advocate for a more sensitive and mental health-friendly climate for prisoners and decrease the stigma of mental illness.

## Figures and Tables

**Table 1:** Background characteristics of study participants (n = 60).

Variables and Response Categories		
**Age** [years] (Mean; SD)	21.9	1.6
	**Frequency (n)**	**%**
**Education**		
None	2	3.3
1st cycle [1st, 2nd, 3rd and 4th grade]	7	11.7
2nd cycle [5th and 6th grade]	19	31.7
3rd cycle [7th, 8th and 9th grade]	29	48.3
Secondary school [10th, 11th and 12th grade]	3	5.0
**Marital status**		
Married/ Domestic partnership	1	1.7
Single	58	96.7
Divorced/widowed/separated	1	1.7
**Professional situation before incarceration**		
Worker	17	28.3
Student	23	38.3
Unemployed	20	33.3
**Nationality**		
Cape Verdean	6	10.0
Guinean	9	15.0
Portuguese	40	66.7
Other	5	8.4
**History of chronic diseases (morbidity)**		
Yes	7	11.7
No	52	86.7
Unsure	1	1.7
**Presence or history of SARS-CoV-2 infection**		
Yes	26	43.3
No	34	56.7

**Table 2:** Mental health status, fear, coping, and health-related perceptions of participants (n = 60).

Variables	Categories	n (%)
**Resilient Coping scores**	Low (4–13)	38 (63.4)
	Medium (14–16)	14 (23.3)
	High (17–20)	8 (13.3)
**Depression scores**	Normal (0–9)	38 (63.3)
	Mild (10–12)	9 (15.0)
	Moderate (13–20)	7 (11.7)
	Severe (21–27)	6 (10.0)
	Extremely severe (28–42)	0
**Anxiety scores**	Normal (0–6)	37 (61.7)
	Mild (7–9)	3 (5.0)
	Moderate (10–14)	8 (13.3)
	Severe (15–19)	5 (8.3)
	Extremely severe (20–42)	7 (11.7)
**Stress scores**	Normal (0–10)	15 (25.0)
	Mild (11–18)	29 (48.3)
	Moderate (19–26)	9 (15.0)
	Severe (27–34)	7 (11.7)
	Extremely severe (35–42)	0
**Perception of global health**	Bad	0
	Fair	6 (10.2)
	Good	19 (32.2)
	Very Good	25 (42.4)
	Excellent	9 (15.3)
**Perception of physical health**	Bad	0
	Fair	6 (10.2)
	Good	18 (30.5)
	Very Good	20 (33.9)
	Excellent	15 (25.4)
**Perception of mental health**	Bad	0
	Fair	9 (15.3)
	Good	29 (47.5)
	Very Good	18 (30.5)
	Excellent	4 (6.8)
**Variables**		**mean ± SD [min-max]**
**Fear of COVID-19 (FCV-19S)**		17.38 ± 4.80 [7,8,9,10,11,12,13,14,15,16,17,18,19,20,21,22,23,24,25,26,27,28,29,30,31,32,33,34,35]
**Perception of mental health**		3.62 ± 0.87 [1,2,3,4,5]
**Perception of physical health**		3.73 ± 0.95 [1,2,3,4,5]
**Perception of global health**		3.27 ± 0.82 [1,2,3,4,5]

**Table 3:** Bivariate correlations among variables (n = 60).

Variables	[min.–max.]	Mean	SD	1	2	3	4
**1. Depression**	0–42	9.05	8.54	-			
**2. Anxiety**	0–42	9.92	7.67	0.644 *	-		
**3. Stress**	0–42	16.13	8.43	0.740 *	0.681 *	-	
**4. Fear of COVID-19**	7–35	17.38	4.80	0.442 *	0.579 *	0.579 *	-
**5. Resilient coping**	4–20	12.73	3.24	−0.017	−0.130	−0.215	−0.052

* *p* < 0.001.

**Table 4:** Hierarchical multiple regression analysis predicting fear of COVID-19 (n = 60).

Predictors	Model I			Model II			Model III		
	B	SE	*β*	B	SE	*β*	B	SE	*β*
Education	0.972	1.246	0.102	0.545	1.308	0.057	0.174	0.207	0.107
Age (years)	0.634	0.330	0.215 *	0.417	0.369	0.142 *	0.094	0.054	0.188 *
Professional situation	−0.321	0.761	−0.053	−0.524	0.810	−0.086	−0.037	0.126	−0.036
Nationality	−0.468	1.327	−0.046	−1.577	1.408	−0.156	−0.089	0.220	−0.052
Presence/History of SARS-CoV-2 Infection	-			−0.856	0.952	−0.096	−0.562	0.752	−0.156
History of chronic diseases	-			−1.056	1.803	−0.078	−2.740	1.755	−0.202
Perception of global health	-			−1.027	0.933	−0.185	−0.159	0.139	−0.168
Perception of physical health	-			1.110	0.804	0.221	0.131	0.120	0.154
Perception of mental health	-			−2.596	0.689	−0.443 **	−0.170	0.153	−0.171 *
DASS-21—Depression	-			-			−0.238	0.230	−0.178
DASS-21—Anxiety	-			-			0.531	0.218	0.324 *
DASS-21—Stress	-			-			0.510	0.267	0.396 *
Resilient Coping	-			-			0.191	0.137	0.190
**R^2^**	0.071			0.273			0.497		
**F**	1.562 *			2.090 ***			3.492 ***		

B—unstandardized regression coefficient; SE—Standard Error; *β*—standardized regression coefficient; * *p* < 0.05, ** *p* < 0.01, *** *p* < 0.001.

## Data Availability

All data generated or analyzed during this study are included in this article. This article is based on the first author’s master’s dissertation in Mental Health and Psychiatric Nursing at the School of Health Sciences—Polytechnic University of Leiria.

## References

[1] Lai C.C., Shih T.P., Ko W.C., Tang H.J., Hsueh P.R. (2020). Severe acute respiratory syndrome coronavirus 2 (SARS-CoV-2) and coronavirus disease-2019 (COVID-19): The epidemic and the challenges. Int. J. Antimicrob. Agents.

[2] Yang H., Thompson J.R. (2020). Fighting COVID-19 outbreaks in prisons. BMJ.

[3] Esposito M., Salerno M., Di Nunno N., Ministeri F., Liberto A., Sessa F. (2022). The Risk of COVID-19 Infection in Prisons and Prevention Strategies: A Systematic Review and a New Strategic Protocol of Prevention. Healthcare.

[4] Saloner B., Parish K., Ward J.A., DiLaura G., Dolovich S. (2020). COVID-19 Cases and Deaths in Federal and State Prisons. JAMA.

[5] Bick J.A. (2007). Infection control in jails and prisons. Clin. Infect. Dis..

[6] LeMasters K., Ranapurwala S., Maner M., Nowotny K.M., Peterson M., Brinkley-Rubinstein L. (2022). COVID-19 community spread and consequences for prison case rates. PLoS ONE.

[7] Akiyama M.J., Spaulding A.C., Rich J.D. (2020). Flattening the Curve for Incarcerated Populations—COVID-19 in Jails and Prisons. N. Engl. J. Med..

[8] Wildeman C., Andersen L.H. (2020). Solitary confinement placement and post-release mortality risk among formerly incarcerated individuals: A population-based study. Lancet Public Health.

[9] Stewart A., Cossar R., Stoové M. (2020). The response to COVID-19 in prisons must consider the broader mental health impacts for people in prison. Aust. N. Z. J. Psychiatry.

[10] World Health Organization (2020). Preparedness, Prevention and Control of COVID-19 in Prisons and Other Places of Detention.

[11] de Sousa G.M., Tavares V., de Meiroz Grilo M., Coelho M., de Lima-Araújo G.L., Schuch F.B., Galvão-Coelho N.L. (2021). Mental Health in COVID-19 Pandemic: A Meta-Review of Prevalence Meta-Analyses. Front. Psychol..

[12] Ornell F., Schuch J.B., Sordi A.O., Kessler F.H. (2020). Pandemia de medo e Covid-19: Impacto na saúde mental e possíveis estratégias. Rev. Debates Psychiatry.

[13] Tekir Ö. (2022). The relationship between fear of COVID-19, psychological well-being and life satisfaction in nursing students: A cross-sectional study. PLoS ONE.

[14] Ahorsu D.K., Lin C.Y., Imani V., Saffari M., Griffiths M.D., Pakpour A.H. (2022). The Fear of COVID-19 Scale: Development and Initial Validation. Int. J. Ment. Health Addict..

[15] Silva Junior F.J., Sales J.C., Monteiro C.F., Costa A.P., Campos L.R., Miranda P.I., Monteiro T.A., Lima R.A., Lopes-Junior L.C. (2020). Impact of COVID-19 pandemic on mental health of young people and adults: A systematic review protocol of observational studies. BMJ Open.

[16] Johnson L., Gutridge K., Parkes J., Roy A., Plugge E. (2021). Scoping review of mental health in prisons through the COVID-19 pandemic. BMJ Open.

[17] Crowley D., Cullen W., O’Donnell P., Van Hout M.C. (2020). Prison and opportunities for the management of COVID-19. BJGP Open.

[18] Pedrosa A.L., Bitencourt L., Fróes A.C., Cazumbá M.L., Campos R.G., de Brito S.B., Simões e Silva A.C. (2020). Emotional, Behavioral, and Psychological Impact of the COVID-19 Pandemic. Front. Psychol..

[19] Thekkumkara S.N., Jagannathan A., Muliyala K.P., Murthy P. (2022). Psychosocial Interventions for Prisoners with Mental and Substance Use Disorders: A Systematic Review. Indian J. Psychol. Med..

[20] Kim H., Hughes E., Cavanagh A., Norris E., Gao A., Bondy S.J., McLeod K.E., Kanagalingam T., Kouyoumdjian F.G. (2022). The health impacts of the COVID-19 pandemic on adults who experience imprisonment globally: A mixed methods systematic review. PLoS ONE.

[21] Abrunhosa Gonçalves R., Andrade J., Gabrielli F., Irtelli F. (2021). Mental Health Issues during COVID-19 Pandemic in Portuguese Prisons. Anxiety, Uncertainty, and Resilience During the Pandemic Period—Anthropological and Psychological Perspectives.

[22] World Prison Brief World Prison Brief Data: Portugal. World Prison Brief. https://www.prisonstudies.org/country/portugal.

[23] Redondo S., Gonçalves R.A., Nistal J., Soler C., Moreira J., Andrade J., Andrés-Pueyo A. (2020). Corrections and Crime in Spain and Portugal during the Covid-19 Pandemic: Impact, Prevention and Lessons for the Future. Vict. Offenders.

[24] Rodrigues A., Antunes M., Fidalgo S., Pinto I., Ishiy K. (2022). The Impact of the COVID-19 Pandemic on the Imposition and Implementation of Alternatives to Prison and Preparation of Individuals for Release in Portugal.

[25] Biggs A., Brough P., Drummond S. (2017). Lazarus and Folkman’s Psychological Stress and Coping Theory.

[26] Glanz K., Rimer B.K., Viswanath K. (2015). Health Behavior: Theory, Research and Practice.

[27] Pattavina A., Palmieri M. (2020). Fears of COVID-19 Contagion and the Italian Prison System Response. Vict. Offenders.

[28] Hewson T., Green R., Shepherd A., Hard J., Shaw J. (2021). The effects of COVID-19 on self-harm in UK prisons. BJPsych Bull..

[29] Konaszewski K., Niesiobędzka M., Surzykiewicz J. (2021). Resilience and mental health among juveniles: Role of strategies for coping with stress. Health Qual Life Outcomes.

[30] Sorge A., Bassanini F., Zucca J., Saita E. (2021). “Fear can hold you, hope can set you free”. Analysis of Italian prisoner narrative experience of the COVID-19 pandemic. Int. J. Prison. Health.

[31] von Elm E., Altman D.G., Egger M., Pocock S.J., Gøtzsche P.C., Vandenbroucke J.P., STROBE Initiative (2008). The Strengthening the Reporting of Observational Studies in Epidemiology (STROBE) statement: Guidelines for reporting observational studies. J. Clin. Epidemiol..

[32] Direção Geral de Reinserção e Serviços Prisionais Estabelecimento Prisional de Leiria (Jovens). https://dgrsp.justica.gov.pt/Justi%C3%A7a-de-adultos/Penas-e-medidas-privativas-de-liberdade/Estabelecimentos-prisionais/%C3%81rea-territorial-alargada-do-tribunal-de-execu%C3%A7%C3%A3o-de-penas-de-Coimbra/Estabelecimento-Prisional-de-Leiria-Jovens.

[33] Lovibond P., Lovibond S. (1994). The structure of negative emotional states: Comparison of the Depression Anxiety Stress Scales (DASS) with the Beck Depression and Anxiety Inventories. Med. Biol. Eng. Comput..

[34] Apóstolo J.L., Mendes A.C., Azeredo Z.A. (2006). Adaptation to Portuguese of the Depression, Anxiety and Stress Scales (DASS). Rev. Lat. Am. Enfermagem..

[35] Laranjeira C., Dixe M.A., Valentim O., Charepe Z., Querido A. (2021). Mental Health and Psychological Impact during COVID-19 Pandemic: An Online Survey of Portuguese Higher Education Students. Int. J. Environ. Res. Public Health.

[36] Soares F.R., Afonso R.M., Martins A.P., Pakpour A.H., Rosa C.P. (2022). The fear of the COVID-19 Scale: Validation in the Portuguese general population. Death Stud..

[37] Sinclair V., Wallston K. (2004). The development and psychometric evaluation of the Brief Resilient Coping Scale. Assessment.

[38] Pais Ribeiro J., Morais R. (2010). Adaptação portuguesa da escala breve de coping resiliente. Psicol. Saúde Doenças.

[39] Cicchetti D.V. (1994). Guidelines, Criteria, and Rules of Thumb for Evaluating Normed and Standardized Assessment Instruments in Psychology. Psychol. Assess..

[40] Tabachnick B.G., Fidell L.S. (2007). Using Multivariate Statistics.

[41] Kołodziej K., Kurowska A., Majda A. (2021). Intensity of perceived stress and control of anger, anxiety and depression of people staying in Polish penitentiary institutions. Int. J. Prison. Health.

[42] Butcher E., Packham C., Williams M., Miksza J., Kaul A., Khunti K., Morriss R. (2021). Screening male prisoners for depression and anxiety with the PHQ-9 and GAD-7 at NHS Healthcheck: Patterns of symptoms and caseness threshold. BMC Psychiatry.

[43] Salari N., Hosseinian-Far A., Jalali R., Vaisi-Raygani A., Rasoulpoor S., Mohammadi M., Rasoulpoor S., Khaledi-Paveh B. (2020). Prevalence of stress, anxiety, depression among the general population during the COVID-19 pandemic: A systematic review and meta-analysis. Global. Health.

[44] Valizadeh R., Veisani Y., Delpisheh A., Kikhavani S., Sohrabnejad A. (2017). Major depression and psychiatric disorders in Iranian prisoners based on a clinical interview: A systematic review and meta-analysis. Shiraz E Med. J..

[45] Birkie M., Necho M., Tsehay M., Gelaye H., Beyene A., Belete A., Asmamaw A., Tessema Z.T., Bogale K., Adane M. (2022). Depressive, Anxiety Symptom Frequency and Related Factors Among Prisoners During the COVID-19 Pandemic in Northeastern Ethiopia, a Cross-Sectional Study. Front. Psychiatry.

[46] Bedaso A., Ayalew M., Mekonnen N., Duko B. (2020). Global Estimates of the Prevalence of Depression among Prisoners: A Systematic Review and Meta-analysis. Depress. Res. Treat..

[47] Huang Y., Wu R., Wu J., Yang Q., Zheng S., Wu K. (2020). Psychological resilience, self-acceptance, perceived social support and their associations with mental health of incarcerated offenders in China. Asian J. Psychiatr..

[48] Moore K.E., Siebert S., Brown G., Felton J., Johnson J. (2021). Stressful life events among incarcerated women and men: Association with depression, loneliness, hopelessness, and suicidality. Health Justice.

[49] Shah S.M., Mohammad D., Qureshi M.F., Abbas M.Z., Aleem S. (2021). Prevalence, Psychological Responses and Associated Correlates of Depression, Anxiety and Stress in a Global Population, During the Coronavirus Disease (COVID-19) Pandemic. Community Ment. Health J..

[50] Suhomlinova O., Ayres T.C., Tonkin M.J., O’Reilly M., Wertans E., O’Shea S.C. (2021). Locked up While Locked Down: Prisoners’ Experiences of the COVID-19 Pandemic. Br. J. Criminol..

[51] Barnert E.S. (2020). COVID-19 and Youth Impacted by Juvenile and Adult Criminal Justice Systems. Pediatrics.

[52] Broche-Pérez Y., Fernández-Fleites Z., Fernández-Castillo E., Jiménez-Puig E., Vizcaíno-Escobar A.E., Ferrer-Lozano D.M., Martínez-Rodríguez L., Martín-González R. (2021). Anxiety, Health Self-Perception, and Worry about the Resurgence of COVID-19 Predict Fear Reactions Among Genders in the Cuban Population. Front. Glob. Womens Health.

[53] Coelho C.M., Suttiwan P., Arato N., Zsido A.N. (2020). On the Nature of Fear and Anxiety Triggered by COVID-19. Front. Psychol..

[54] Carleton R.N. (2016). Fear of the unknown: One fear to rule them all?. J. Anxiety Disord..

[55] Hewson T., Shepherd A., Hard J., Shaw J. (2020). Effects of the COVID-19 pandemic on the mental health of prisoners. Lancet Psychiatry.

[56] Satici B., Saricali M., Satici S.A., Griffiths M.D. (2022). Intolerance of uncertainty and mental wellbeing: Serial mediation by rumination and fear of COVID-19. Int. J. Ment. Health Addict..

[57] Kuang J., Ashraf S., Das U., Bicchieri C. (2020). Awareness, Risk Perception, and Stress during the COVID-19 Pandemic in Communities of Tamil Nadu, India. Int. J. Environ. Res. Public Health..

[58] Andrade E.F., Pereira L.J., Oliveira A.P.L., Orlando D.R., Alves D.A.G., Guilarducci J.S., Castelo P.M. (2022). Perceived fear of COVID-19 infection according to sex, age and occupational risk using the Brazilian version of the fear of COVID-19 scale. Death Stud..

[59] Bäuerle A., Teufel M., Musche V., Weismüller B., Kohler H., Hetkamp M., Dörrie N., Schweda A., Skoda E.M. (2020). Increased generalized anxiety, depression and distress during the COVID-19 pandemic: A cross-sectional study in Germany. J. Public Health.

[60] Bakioğlu F., Korkmaz O., Ercan H. (2021). Fear of COVID-19 and positivity: Mediating role of intolerance of uncertainty, depression, anxiety, and stress. Int. J. Ment. Health Addict..

[61] Khalaf O.O., Abdalgeleel S.A., Mostafa N. (2022). Fear of COVID-19 infection and its relation to depressive and anxiety symptoms among elderly population: Online survey. Middle East. Curr. Psychiatry.

[62] Simpson P.L., Guthrie J., Jones J., Butler T. (2021). Identifying research priorities to improve the health of incarcerated populations: Results of citizens’ juries in Australian prisons. Lancet Public Health.

[63] Simpson A.I., Gerritsen C., Maheandiran M., Adamo V., Vogel T., Fulham L., Kitt T., Forrester A., Jones R.M. (2022). A Systematic Review of Reviews of Correctional Mental Health Services Using the STAIR Framework. Front. Psychiatry.

[64] Maycock M. (2022). ‘COVID-19 has caused a dramatic change to prison life’. Analysing the impacts of the COVID-19 pandemic on the pains of imprisonment in the Scottish Prison Estate. Br. J. Criminol..

[65] Ricciardelli L.A., King E., Broadley M. (2022). COVID-19, Mental Illness, and Incarceration in the United States: A Systematic Review, 2019–2021. Soc. Sci..

[66] Kothari R., Sparrow J., Henshall J., Buchan D., Kemp J., Owen A., Blakeman I., Sarkissian N. (2022). Locked Up and Locked Down: How the COVID-19 Pandemic has Impacted the Mental Health of Male Prisoners and Support Staff. J. Mens. Health.

[67] Berberoglu A., Dinler A. (2020). The mediator role of communication about COVID-19 on the relationship between exaggeration of media and generated fear: Case of North Cyprus. J. Clin. Exp. Investig..

